# Primary Hepatic Lymphoma in a Patient with Rheumatoid Arthritis Treated with Methotrexate

**DOI:** 10.1155/2014/460574

**Published:** 2014-12-24

**Authors:** Goichi Tatsumi, Naoya Ukyo, Hirokazu Hirata, Mitsuru Tsudo

**Affiliations:** Department of Hematology, Osaka Red Cross Hospital, 5-30 Fudegasaki-cho, Tennoji-ku, Osaka 543-8555, Japan

## Abstract

Primary hepatic lymphoma (PHL) has rarely been reported in patients with immunosuppression. We herein describe a case of Epstein-Barr virus- (EBV-) positive PHL in a 67-year-old Japanese woman receiving methotrexate (MTX) treatment for rheumatoid arthritis (RA). The patient, who had been receiving MTX therapy for more than 6 years, presented with low-grade fever and abdominal pain. Initial laboratory tests showed mildly elevated liver enzymes with normal levels of alpha-fetoprotein and carcinoembryonic antigen, and computed tomography scans revealed multiple hepatic tumors with no lymph-node swelling. Examination of liver specimens obtained via ultrasonography-guided needle biopsy indicated EBV-positive diffuse large B cell lymphoma; therefore, she was diagnosed with PHL. MTX was discontinued, and she was carefully monitored thereafter owing to the prolonged history of MTX administration for RA. Rapid progression of PHL was observed; therefore 10 days after the PHL diagnosis, she received 6 cycles of R-THP-COP (rituximab, cyclophosphamide, pirarubicin, vincristine, and prednisolone) therapy and achieved complete remission for more than 1 year. Although MTX-associated lymphoproliferative disorders often show remission after withdrawal of MTX, early diagnosis and treatment are essential for PHL in patients with RA treated with MTX, because of the aggressive nature of the disease.

## 1. Introduction

Hepatic involvement in non-Hodgkin lymphoma (NHL) is a relatively common presentation of extranodal disease spread. However, primary hepatic lymphoma (PHL) is rare, accounting for less than 1% of all extranodal lymphomas [1]. According to previous reports, PHL tends to occur in patients with hepatitis C virus (HCV) infection or immunosuppressive disorders, such as systemic lupus erythematosus and acquired immune deficiency syndrome, and in those receiving immunosuppressive treatment [1, 2]. Methotrexate- (MTX-) associated lymphoproliferative disorders (LPDs) are generally lymphoid proliferation or lymphomas occurring in immunosuppressed patients receiving MTX. Although extranodal NHL is quite common, occurring in up to 50% of all MTX-LPD cases, PHL in patients with rheumatoid arthritis (RA) treated with MTX has rarely been reported [3, 4]. Herein we describe a case of primary diffuse large B-cell lymphoma (DLBCL) of the liver in a patient with RA who had been treated with MTX for approximately 6 years.

## 2. Case Report

A 67-year-old woman presented with a 1-week history of low-grade fever and intermittent pain in the right upper quadrant of the abdomen. The patient's medical history revealed that she had seronegative RA for 38 years; she had been undergoing treatment with MTX and prednisolone for more than 6 years. As her adherence to RA treatment had been quite poor before starting MTX and prednisolone at the previous clinic, her joints of fingers and toes were severely deformed. The dose of MTX was 6 mg/week for 6 years and prednisolone was slowly tapered to 0.5 mg/day, and she had never received antitumor necrosis factor (TNF) therapy. Lately her symptoms of joints were improved and were stable.

**Figure 1:**
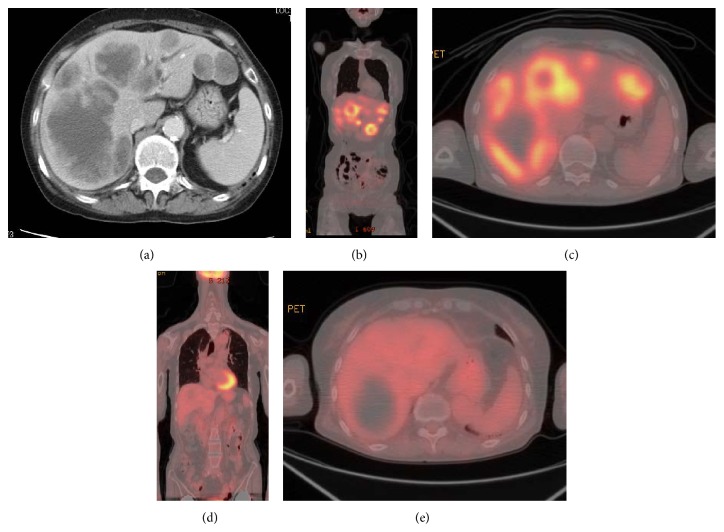
Enhanced CT images and positron emission tomography/computed tomography (PET/CT) before ((a)–(c)) and after ((d), (e)) chemotherapy.

**Figure 2:**
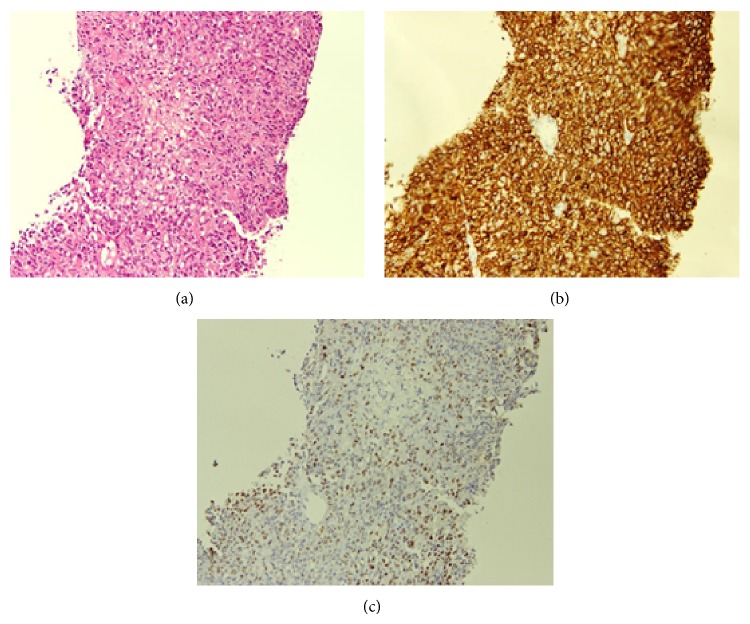
Histopathology of the liver biopsy shows (a) infiltration of medium-to-large lymphoid cells (hematoxylin and eosin, 100x), (b) CD20 positivity (100x), and (c) Epstein-Barr virus-encoded small RNA positivity (100x).

**Figure 3:**
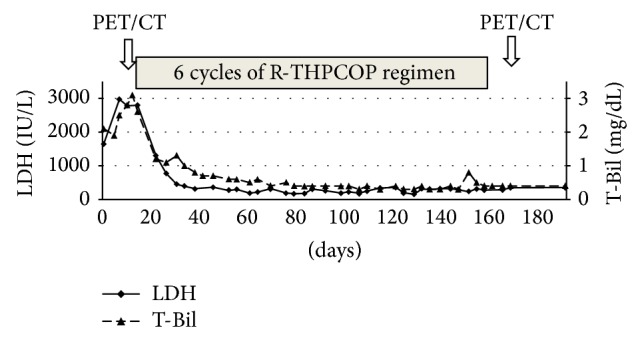
Clinical course of the patient. The *x*-axis presents time (days) from admission to our hospital. LDH: lactate dehydrogenase; T-Bil: total bilirubin; and PET/CT: positron emission tomography/computed tomography.

Physical examination at presentation indicated general fatigue; her temperature was 37°C, and her conjunctiva was neither icteric nor anemic. Her performance status was 2. Abdominal tenderness was observed in the right upper quadrant, without any hepatomegaly. The spleen and superficial lymph nodes were not palpable. Her joints of fingers and toes were severely deformed, but she had no pain, tenderness, or swelling in her joints. Laboratory studies on admission showed the following results: total bilirubin content, 2.1 mg/dL (normal range: 0.2–1.0 mg/dL); aspartate aminotransferase level, 153 IU/L (8–38 IU/L); alanine aminotransferase level, 92 IU/L (4–44 IU/L); lactate dehydrogenase (LDH) level, 1646 IU/L (120–260 IU/L); and alkaline phosphatase level, 1991 IU/L (104–338 IU/L). Complete blood counts were normal. Serum interleukin-2 receptor was elevated at 3720 U/mL (145–519 U/mL), but the levels of other tumor markers, such as alpha-fetoprotein (AFP) and carcinoembryonic antigen (CEA), were normal. Serology was negative for hepatitis B virus (HBV) surface antigen, HCV, and human immunodeficiency virus. Computed tomography (CT) revealed multiple hypodense nodules with irregular margins, ranging from 8 to 65 mm, throughout the whole liver (Figure 1(a)). Positron emission tomography (PET)/CT with [^18^F]-fluorodeoxyglucose (FDG) was performed to further investigate the hepatic lesions and to determine the extent of disease. These lesions demonstrated ring-like hypermetabolic foci, with a maximum standard uptake value of 16.6. No other foci with abnormal FDG uptake were detected elsewhere in the body (Figures 1(b) and 1(c)). A diagnostic ultrasonography-guided fine-needle biopsy was also performed. Histological examination showed diffuse infiltration of medium-to-large atypical lymphoid cells, with necrosis. The cells were positive for CD20 via immunohistochemical analysis and for Epstein-Barr virus- (EBV-) encoded small RNA via in situ hybridization (Figures 2(a)–2(c)). Flow cytometry analysis showed a pathological B-cell phenotype with positivity for CD19 and CD20 and negativity for CD5 and CD10. Cytogenetic analysis detected a complicatedly abnormal karyotype. Bone marrow examination showed no infiltration by the lymphoma. As no other foci of lymphoma were observed, a final diagnosis of MTX-LPD with the features of DLBCL was established. Subsequently, the patient's liver function continued to deteriorate rapidly, despite MTX discontinuation. She then received 6 cycles of R-THP-COP (rituximab, cyclophosphamide, pirarubicin, vincristine, and prednisolone) therapy. Soon after chemotherapy was initiated, her symptoms improved and laboratory measurements of liver function (including LDH levels and total bilirubin content) gradually decreased and returned to normal within 4 weeks (Figure 3). Follow-up FDG-PET/CT was performed 5 weeks after completion of chemotherapy, no FDG uptake by the liver lesions was detected, and only a hypodense area was observed on the CT scan, thus confirming necrotic changes (Figures 1(d) and 1(e)). During the subsequent 1-year follow-up, our patient showed no symptoms or signs of recurrent disease. She also started 500 mg of salazosulfapyridine for RA after the chemotherapy and she experienced no deterioration in her joints.

## 3. Discussion

PHL was first reported in 1965 by Ata and Kamel [5] and is defined as an extranodal lymphoma confined to the liver [6]. Although secondary involvement of the liver is relatively common in NHL (21% of all advanced cases), PHL is extremely rare, accounting for only 0.4% of all extranodal NHL and 0.016% of all NHL cases [7]. PHL is more frequent in men, and the usual age at presentation is approximately 50 years [1, 2, 6]. The most common histological type of PHL is DLBCL (approximately 46–68% of all cases) [6]. The initial presentations of PHL are generally nonspecific with hepatomegaly abdominal pain (right upper quadrant or epigastric pain), weight loss, and fever being the most common symptoms. The results of liver function tests are typically abnormal. Elevated LDH and ALP levels, accompanied by normal serum AFP and CEA levels, as in our case, have been reported to be important diagnostic features [1, 2, 6].

The etiology of PHL is largely unknown, although many cases are reportedly associated with HBV and HCV infection, prior to EBV infection, cirrhosis, immunosuppressive therapy, and autoimmune diseases [4]. Our patient's test results were negative for the hepatitis viruses, but she had a long history of low-dose MTX therapy for RA. MTX is an effective immunosuppressive agent that is widely prescribed for patents with RA and the resulting immunosuppressive state might be the basis of LPD or lymphoma development. The dose of MTX, which was low in this case because the patient was a very petite woman and there was a dosing restriction (up to 8 mg/week) until 2011 in Japan, was reported to be an independent risk factor for LPD onset in RA patients recently [8]. On the other hand, EBV is frequently associated with LPD development, and EBV positivity is observed in approximately 30–50% of all LPD cases diagnosed in patients with RA treated with MTX [3, 9]. Furthermore, approximately 40–50% of the reported MTX-LPD cases have involved extranodal sites, such as the gastrointestinal tract, skin, lungs, kidneys, and soft tissues [10, 11]. However, to our knowledge, only two cases of MTX-LPD occurring exclusively in the liver have been reported in the English literature [4].

Imaging studies, both ultrasonography and CT, are helpful for diagnosis. PHL exhibits characteristic imaging features, which can be classified into the following three morphologic patterns: solitary mass, multiple nodules, and diffuse infiltration. Diffuse infiltration type is rare and more commonly seen in Chinese patients [2, 12]. Previous reports have shown that PHL is more likely present as a solitary mass (55–60%) than as multiple lesions (35–40%) [1, 4, 6, 7]. Our patient's CT scans featured multiple hypodense lesions in the liver, which was indicative of hepatic lymphoma. However, it is often difficult to distinguish hepatic lymphoma from hepatocarcinoma or gastrointestinal tract metastasis, because there are no reported diagnostic features of hepatic lymphoma [13]. Recently, functional imaging with FDG-PET/CT has been increasingly utilized for accurate disease staging and therapeutic response evaluation [14–17]. In our case, the posttreatment FDG-PET/CT scan showed no FDG uptake by the liver lesions, although the CT scan demonstrated relatively smaller but persistent hypodense areas, which was indicative of necrotic changes rather than residual disease.

In order to establish a diagnosis, it is essential to obtain liver specimens by using percutaneous fine needle aspiration (FNA), laparoscopy, or laparotomy. In recent years, minimally invasive image-guided FNA has become popular. Combined immunohistochemistry and flow cytometry analyses of FNA samples could be used to establish a safe, rapid, and accurate diagnosis despite a relatively limited amount of specimen [18, 19]. In our case, surgical biopsy options, such as laparoscopy or laparotomy, were not selected considering the patient's age, performance status, and general medical condition; therefore, percutaneous FNA was performed instead. We were then able to rapidly diagnose the patient with PHL and provide appropriate treatment, without encountering any postoperative complications.

PHL is traditionally considered an aggressive disease with a poor prognosis and its optimal treatment has not been defined [20]. Available therapeutic options include surgery, chemotherapy, radiotherapy, or a multimodality treatment. Surgery, alone or in combination with other modalities, has been reported as the therapy of choice for resectable PHLs; however, it was not feasible in our patient because of the presence of multiple lesions. Current literature favors combination chemotherapy as the frontline treatment owing to its noninvasive nature and improved survival outcomes [18, 21]. The successful outcome of our case was indeed supportive of this notion. Additionally, Page et al. [1] previously reported an overall 5-year survival of 83% in 24 patients treated with multidrug chemotherapy only.

MTX-LPD generally has a good prognosis and approximately half of MTX-LPD patients showed disease regression after discontinuation of MTX [3, 9]. In addition, EBV-positive MTX-LPD cases tend to show regression after MTX withdrawal more often when compared with EBV-negative cases [9]. Therefore, a period of careful observation awaiting spontaneous remission after MTX withdrawal is recommended for the treatment of MTX-LPD [9, 22]. However, as disease recurrence is common, it may be treated successfully using standard chemotherapy [23]. In the present case, we also discontinued MTX and observed the patient carefully without treatment, but her condition deteriorated rapidly in approximately 10 days. Therefore, chemotherapy was quickly initiated prior to fulminant liver failure, and the patient fortunately achieved a complete response.

In conclusion, although PHL is rare in patients with RA treated with MTX, it is one of the most aggressive forms of MTX-LPD. While MTX discontinuation and careful observation are the standard options for management of MTX-LPD, early diagnosis and treatment of PHL are essential in patients with RA treated with MTX, because it is often difficult to induce treatment after the rapid progression of hepatic failure. Our experience in this case indicated that early detection of the disease might offer satisfactory results with standard chemotherapy.
